# Carcinoma-Associated Fibroblasts Are a Promising Therapeutic Target

**DOI:** 10.3390/cancers5010149

**Published:** 2013-01-31

**Authors:** Shinsaku Togo, Urszula M. Polanska, Yoshiya Horimoto, Akira Orimo

**Affiliations:** 1 Department of Respiratory Medicine, Juntendo University School of Medicine, Tokyo 113-8412, Japan; 2 CR-UK Stromal-Tumour Interaction Group, Paterson Institute for Cancer Research, The University of Manchester, Wilmslow Road, Manchester M20 4BX, UK; 3 Department of Pathology and Oncology, Juntendo University School of Medicine, Tokyo 113-8412, Japan; 4 Atopy Research Centre, Juntendo University School of Medicine, Tokyo 113-8412, Japan; 5 Department of Breast Oncology, Juntendo University School of Medicine, Tokyo 113-8412, Japan

**Keywords:** carcinoma-associated fibroblasts (CAFs), tumour microenvironment, stromal-tumour interaction, tumour metastasis, CAF-targeted cancer therapy, innate drug resistance

## Abstract

Human carcinomas frequently exhibit significant stromal reactions such as the so-called “desmoplastic stroma” or “reactive stroma”, which is characterised by the existence of large numbers of stromal cells and extracellular matrix proteins. Carcinoma-associated fibroblasts (CAFs), which are rich in activated fibroblast populations exemplified by myofibroblasts, are among the predominant cell types present within the tumour-associated stroma. Increased numbers of stromal myofibroblasts are often associated with high-grade malignancies with poor prognoses in humans. CAF myofibroblasts possess abilities to promote primary tumour development, growth and progression by stimulating the processes of neoangiogenesis as well as tumour cell proliferation, survival, migration and invasion. Moreover, it has been demonstrated that CAFs serve as a niche supporting the metastatic colonisation of disseminated carcinoma cells in distant organs. Their contribution to primary and secondary malignancies makes these fibroblasts a potential therapeutic target and they also appear to be relevant to the development of drug resistance and tumour recurrence. This review summarises our current knowledge of tumour-promoting CAFs and discusses the therapeutic feasibility of targeting these cells as well as disrupting heterotypic interactions with other cell types in tumours that may improve the efficacy of current anti-tumour therapies.

## 1. Introduction

Tumours are a complex tissue composed of carcinoma cells, various types of stromal cells and dense extracellular matrix (ECM). Recent studies have indicated the tumour-promoting roles of stromal cells, as exemplified by vascular cells, immune cells, fibroblasts, myofibroblasts, adipocytes and bone marrow-derived progenitors [1,2,3,4,5,6]. Importantly, the relevance of these stromal cells to failure of systemic drug delivery to tumours and the development of drug resistance has also been indicated [7,8,9,10,11]. The identification of cellular and molecular targets abrogating stromal-tumour cell interactions and thus attenuating tumourigenesis is currently one of the most important subjects in translational oncology. Achieving this goal is essential for increasing the efficacy of conventional therapies in combination with the stroma-based therapeutic approaches [12,13].

Considerable numbers of CAFs are frequently observed within the tumour-associated stroma of various human cancers, including those of the breast, prostate, lung, colon and pancreas [14,15]. These cells contain distinct subpopulations of activated fibroblasts exemplified by myofibroblasts positive for α-smooth muscle actin (α-SMA) [14,16,17,18]. CAFs have been also shown to originate from various sources, such as local resident tissues and bone marrow [19,20,21,22], indicating their diverse cellular origins.

In addition to the ability of CAFs to promote neoangiogenesis and tumour growth [23,24,25,26,27,28], recent reports have indicated emerging roles for these cells in the modulation of cancer stem cell (CSC) traits and in the formation of metastases [29,30,31,32]. Interaction of CAFs with carcinoma cells and other stromal cells has thus been demonstrated to be crucial for the development of aggressive tumours during the course of cancer progression. CAFs facilitate the conversion of otherwise incipient tumour cells into highly malignant cells which can spread to and infiltrate distant organs, ostensibly due to the acquisition of invasive and metastatic phenotypes. However, molecular mechanisms underlying this CAF-stimulated malignant conversion and their potential utility in designing relevant therapeutic interventions, require further studies. In this review, we highlight the biological significance of tumour-promoting functions of CAFs and discuss their potential therapeutic applications.

## 2. Distinct Cellular Origins of CAFs and Their Activated Phenotypes

Both activated and non-activated fibroblasts comprise CAF populations in the tumour-associated stroma. Activated stromal fibroblast populations in these cells have often been identified by the expression of several markers including α-SMA [18,33], tenascin-C (TN-C) [34], periostin (POSTN) [30,35], NG2 chondroitin sulfate proteoglycan (NG2) [21], platelet derived growth factor receptor alpha/beta (PDGFRα/β) [24,36] and fibroblast activation protein (FAP) [14,37] (Table 1). Markers, such as vimentin [14], fibronectin [38], type I collagen [14], prolyl 4-hydroxylase [38], fibroblast surface protein [38] and fibroblast specific protein-1 (FSP-1)/S100A4 [14,16] are also often used to detect various types of mesenchymal cells including CAFs. Moreover, immunohistochemical analysis has shown overlapped expression of NG2 and PDGFRβ on α-SMA-positive myofibroblasts in the autochthonous mouse pancreatic cancer and the human breast tumour xenograft [21]. FSP-1 was, however, detected mainly on non-myofibroblastic populations in tumour, suggesting distinct fibroblast populations present in CAFs [21].

**Table 1:** Markers detecting activated fibroblast populations in CAFs. CAFs consist of both activated and non-activated fibroblasts in the tumour-associated stroma. Several different markers, such as α-SMA [18,33], tenascin-C (TN-C) [34], periostin (POSTN) [30,35], neuron-glial antigen2 (NG2) [21], PDGFRα/β [24,36], fibroblast activated protein (FAP) [14,37], palladin [39] and podoplanin [40] are reported to be useful for detecting activated stromal fibroblast populations in CAFs.

Markers for activated stromal fibroblasts in tumours	
α-SMA [18,33]	FAP [14,37]
TN-C [34]	Palladin [39]
POSTN [30,35]	Podoplanin [40]
NG2 [21]	
PDGFRα/β [24,36]	

CAFs are also known to originate from heterogeneous cell types including resident fibroblasts, preadipocytes, endothelial cells and bone marrow-derived cells, such as mesenchymal stem cells (MSCs) and fibrocytes [22,41,42]. A recent study further investigated the cellular origins of CAFs using a transgenic mouse model [19]. The latter ubiquitously expressed red fluorescent protein (RFP) but was reconstituted with GFP-positive (GFP^+^) bone marrow prior to subcutaneous implantation of murine ovarian carcinoma cells. Importantly, the majority of FSP-1^+^ and FAP^+^ fibroblasts in the resulting tumour xenografts were shown to derive largely from GFP^+^ bone marrow cells including MSCs. In contrast, α-SMA^+^ and NG2^+^ myofibroblasts in tumour started to develop mainly from RFP^+^ local resident tissue exemplified by the adipose tissue [19].

Taken together, these findings suggest that CAFs are composed of various fibroblast populations with their distinct cellular origins. Continuous infiltration of a tumour mass by these various fibroblast progenitors, and their subsequent transdifferentiation into CAFs, can facilitate the generation and expansion of the so-called desmoplastic, reactive stroma that in turn supports tumour progression. Potential diverse tumour-promoting functions of the distinct fibroblast populations within CAFs remain to be addressed in future studies.

Activated myofibroblasts, which are characterised by the *de novo* production and incorporation of α-SMA into stress fibres, exhibit a greater ability to contract collagen gels *in vitro*. Our previous work demonstrated that the greater ability of CAFs to contract collagen gels in culture correlated significantly with their ability to more effectively promote the growth of co-implanted human breast carcinoma cells in recipient mice [23]. This finding supported the notion that the activated state in these fibroblasts is a prerequisite for eliciting their tumour-promoting ability presumably via gene regulatory networks relevant to inflammation, neoangiogenesis, tumour invasion and metastasis.

Notably, the activated myofibroblastic trait and the tumour-promoting phenotypes of CAFs were stably maintained during their *in vitro* propagation without the ongoing interaction with carcinoma cells [23,38]. Primary myofibroblasts originating from the tissues affected by chronic fibrotic diseases also showed stable activated phenotypes [43,44,45]. Importantly, a recent *in vivo* study employing experimentally generated models of kidney fibrosis demonstrated that DNA methylation involves maintaining the stability of fibroblast activation [46]. Transforming growth factor (TGF)-β1, which is abundant in fibrotic tissues, was shown to induce hypermethylation of the Rasal1 gene, an inhibitor of Ras and a member of the Ras GTPase activating protein family [46]. The resulting inhibition of Rasal1 expression led to continuous hyperactivity of Ras signalling that promoted fibroblast activation and proliferation. Rasal1 hypermethylation was also detected in renal fibroblasts extracted from patients suffering from various fibrotic diseases. Collectively, these findings demonstrated that continuous activation of myofibroblasts during fibrosis is induced and maintained by TGF-β1-induced Rasal1 DNA hypermethylation and the resulting Ras hyperactivity. It remains to be determined, however, whether the activated phenotype of CAFs is mediated by the same epigenetic mechanism.

## 3. The Signalling Pathways Relevant to Tumour-Promoting Phenotypes of CAFs

It has been shown and widely accepted that carcinoma cells and stromal cells co-evolve during the course of tumour progression [38,47]; carcinoma cells can educate the surrounding stromal cells allowing their conversion into activated tumour-supporting cells. Such stromal cells reciprocate in stimulating tumour progression by secreting tumour-promoting growth factors and cytokines. To date, various reports have indicated the importance of several signalling pathways in governing the activated, tumour-promoting ability of CAFs, including those of TGF-β-Smad2/3, CXCL12/stromal cell-derived factor 1 (SDF-1)-CXCR4, interleukin 1β (IL-1β)-NF-κB, platelet-derived growth factor (PDGF)-PDGF receptor (PDGFR), phosphatase and tensin homologue (Pten)-v-ets erythroblastosis virus E26 oncogene homolog 2 (Ets2) and Sonic hedgehog (Shh)-Smoothened (Smo) [24,38,48,49,50].

In addition to others, our study showed that subcutaneous co-implantation of human mammary stromal fibroblasts with breast carcinoma cells into recipient mice resulted in transdifferentiation of these fibroblasts into tumour-promoting CAF myofibroblasts during tumour progression [38]. These experimentally generated CAFs were shown to express upregulated levels of TGF-β and CXCL12, and these cytokines were demonstrated to act in an autocrine fashion through activation of their receptors, TGF-β receptor (TβR) and CXCR4, respectively, expressed by CAFs. The establishment of TGF-β-TβR-Smad2/3 and CXCL12-CXCR4 autocrine signalling loops was responsible for induction and maintenance of the activated, myofibroblastic state and tumour-promoting propensity of experimentally generated CAFs [38].

α-SMA-negative (or negligible) PDGFR-α-positive stromal cells extracted from the neoplastic skin of K14-HPV16 (HPV) transgenic mice were also characterised as CAFs [24]. Their tumour-promoting phenotype was induced by immune cell-secreted IL-1β and was dependent on NF-κB signalling. Inflammatory cytokines (e.g., CXCL1, CXCL2 and CCL2), involved in recruiting tumour-associated macrophages (TAMs), were importantly shown to be produced by these cells to further advance tumourigenesis. These findings revealed the significance of the tumour-promoting roles of CAFs in recruiting TAMs to the tumour [4,24].

Pten is one of the main regulators of PI3K signalling and is a tumour suppressor with lipid and protein phosphatase activity [51,52]. The roles of this protein in stromal fibroblasts were studied using spontaneously developing mammary tumours in MMTV-ErbB2/neu; fibroblast-specific protein 1 (Fsp-1)-Cre; Pten^loxP/loxP^ transgenic mice [48]. In this experimental mouse model, the Pten gene was specifically deleted in FSP-1-positive (FSP-1^+^) cells that form a subpopulation of stromal fibroblasts within CAFs. Loss of Pten expression in FSP-1^+^ CAFs resulted in an accelerated growth rate of ErbB2-positive mammary carcinoma. This was suggested to be a consequence of expansion of the desmoplastic stromal reaction involving numerous infiltrating macrophages. Notably, the Pten-deficient stromal fibroblasts upregulated Ets 2 expression and downregulated miR-320, a negative regulator of Ets 2 expression that contributed to increased mammary tumour growth [53]. Thus, inhibition of Ets2 expression by either its genetic deletion or miR-320 overexpression attenuated the promotion of tumour growth by these fibroblasts [48,53]. Collectively, these findings suggest that Pten expression in FSP-1^+^ stromal fibroblasts serves as a negative regulator of Ets2 expression via miR-320 to inherently constrain mammary tumourigenesis.

Recent studies have also shown that carcinoma cell-derived hedgehog (Hh) ligands induce the Smo signalling pathways in the surrounding stroma in a paracrine fashion [49,54,55]. Hh ligand expression was shown to be restricted to epithelial carcinoma cells, whereas increased expressions of the Patched-1 (PTCH1) receptor and Gli1, a downstream mediator of Smo signalling, were detected in the tumour-associated stroma of mouse pancreatic tumour models [56]. The exposure of cultured pancreatic stellate cells to Shh, one of three Hh ligand proteins, also facilitated their differentiation into myofibroblasts [57]. Notably, inhibition of Hh-Smo signalling in tumour-associated stromal cells upon application of either a Smo chemical inhibitor or a neutralising anti-Hh antibody substantially attenuated growth of colon and pancreatic tumour cells *in vivo* [49,54,55]. Taken together, these findings indicated the importance of Hh-Smo signalling in mediating tumour-promoting stromal-tumour interactions and suggested the relevance of this pathway for developing new cancer therapies.

It is assumed that CAFs sustain their various aforementioned tumour-promoting signalling pathways without an ongoing interaction with carcinoma cells. The persistence of CAF phenotypes is presumably achieved through the acquisition of genetic and/or epigenetic alterations in these cells. Recent studies using genome-wide comparative genomic hybridisation and single-nucleotide polymorphism array analyses indicated that clonal somatic genetic alterations are very rarely detected in tumour stroma present within human cancers [47,58,59,60]. This notion has been supported by several independent studies using tumour-associated stromal regions micro-dissected from fresh frozen human breast and ovarian cancer sections and primary cultured CAFs extracted from human breast and pancreatic tumours [47,58,59,60]. Loss of heterozygosity (LOH) has been reported in stromal regions associated with mammary and ovarian tumours in only one of 35 samples tested [60] and in only one of 25 batches of primary mammary CAFs [58]. In sharp contrast, studies employing formalin-fixed paraffin embedded samples showed a higher frequency of detection of LOH and somatic mutations in tumour-associated stroma, such as that of TP53 tumor suppressor gene in 25.6% and 19.4% of patients with sporadic and hereditary breast cancers, respectively [47,61,62,63]. These conflicting observations of the presence of genetic alterations in formalin-fixed tumour stroma, as well as their absence in fresh frozen samples, warrant further future studies to search for gene signalling networks and potential biomarkers in tumour-associated stroma with direct applications for the development of anti-tumour stroma therapies.

Furthermore, understanding of the consequences of altered profiles of DNA methylation within CAF genomes [64,65], identification of the epigenetically affected genes and their related signalling pathways, that could directly mediate the tumour-promoting phenotypes of CAFs, should be subjects of future intensive research.

## 4. Tumour Invasion-, Metastasis- and CSC-Promoting Signalling from CAFs

In addition to the primary tumour-promoting function of CAFs, recent studies have demonstrated an ability of these cells to serve as a niche for CSCs relevant to the promotion of tumour growth, invasion and metastasis [29,30,66,67,68]. Stromal myofibroblasts in human colorectal carcinomas are reported to produce high levels of hepatocyte growth factor (HGF), which acts via the Met receptor on nearby carcinoma cells in a paracrine fashion [69]. This stromal growth factor has been shown to facilitate activation of the Wnt signalling pathway exemplified by nuclear localisation of β-catenin in carcinoma cells that induces their CSC phenotypes illustrated by the increased sphere-forming and tumour-initiating abilities [69]. This work therefore demonstrated the importance of CAF-driven HGF in the activation of Wnt signalling and thereby in conferring the CSC phenotype in colon carcinoma cells.

CCL2 (also called monocyte chemotactic protein-1: MCP-1) is also a well-known potent chemoattractant for monocytes and other immune cells which migrate to areas of inflammation and tumour. This chemokine, produced by CAFs, was reported to confer invasive and CSC phenotypes on carcinoma cells by acting directly through CCR2 and CCR4 receptors [70,71,72]. CAF-secreted CCL2 also increased the activation of Notch signalling in carcinoma cells via induction of p38 mitogen-activated protein kinase (MAPK) signalling, thereby stimulating mammosphere formation [70]. Moreover, inhibition of CCL2 expression and activity in CAFs using short hairpin RNA and a neutralising antibody attenuated the tumourigenesis of co-implanted human breast carcinoma cells in recipient mice. Collectively, these findings suggest that CCL2-CCR2/4 signalling mediates the crosstalk between CAFs and carcinoma cells and is thus a potential therapeutic target in the suppression of the CAF-induced CSC phenotypes.

Another study also indicated that CAFs are responsible for inducing epithelial to mesenchymal transition (EMT) and the associated CSC phenotypes in human prostate carcinoma cells [66]. Two different activated prostate fibroblast populations, generated by treatment with either recombinant IL-6 or TGF-β, showed induction of fibroblast activation protein (FAP) or α-SMA, respectively, both of which are well-known markers of activated fibroblasts. Media conditioned by these activated fibroblasts indeed stimulated EMT, invasion and prostasphere formation in cultured PC3 prostate carcinoma cells. Induction of matrix metalloproteinases or urokinase-type plasminogen activator (uPA)-uPA receptor signalling in carcinoma cells by either activated fibroblasts also reportedly mediated these aggressive carcinoma cell phenotypes. Moreover, co-implantation of primary prostate CAFs or IL-6-induced activated fibroblasts with prostate carcinoma cells subcutaneously into immunodeficient mice resulted in increases in both the incidence and the growth of tumours. Taken together, these findings raise the possibility that distinct paracrine signalling molecules released by different subpopulations of activated stromal fibroblasts play significant roles in promoting tumour progression by conferring EMT and CSC phenotypes.

Tumour-associated stromal cells interacting with ECM proteins also modulate intracellular adhesions and cell contractility, thereby contributing to the generation of tensile forces in the tumour microenvironment that can promote breast cancer progression [73,74]. It has been demonstrated that caveolin-1 (Cav-1), produced by CAFs, alters the alignment of ECM proteins and promotes stiffness of the tumour stroma, thereby resulting in force-dependent Rho GTPase activation and the stimulation of tumour invasion and metastasis [74]. The contractile forces and proteolytic activity induced by CAFs are also known to remodel ECM to generate tracks for the collective invasion of human squamous cell carcinoma (SCC) cells, which were co-cultured on collagen-Matrigel-coated plates [75]. Rho- and Rho-associated protein kinase (ROCK)-dependent regulation of actomyosin interactions was found to be responsible for this CAF-driven tumour cell migration [75]. Moreover, a recent study indicated that the glycoprotein 130 (gp130)-Janus kinase 1(JAK1)-signal transducer and activator of transcription 3 signalling pathway acts in CAFs and nearby tumour cells in a positive feedback fashion, thereby increasing their actomyosin contractility [76]. This cytokine signalling, in collaboration with ROCK signalling, allowed CAFs to induce the collective invasion of human SCC cells and increase the amoeboid motility of human melanoma cells [76]. These findings therefore support the notion that ECM remodelling by CAFs via cytokine signalling promotes tumour cell invasion by modulating the migratory modes which seemingly depend on the responding cancer cell types.

The colonisation of distant organs by disseminated carcinoma cells is one of the most crucial steps in the multi-step process of tumour metastasis. This metastatic colonisation by cancer cells largely depends on the support provided by the stromal niche, which is created by a unique composition of local stromal environmental factors including ECM periostin (POSTN) and tenascin-C (TN-C) proteins [30,77,78]. Molecular signalling pathways, by which CAFs facilitate the metastatic colonisation of disseminated carcinoma cells, have been explored using a transgenic mouse model. Tumour-derived TGF-β3 was shown to initially induce POSTN production in CAFs within pulmonary metastases [30]. The growth of pulmonary metastases spontaneously arising from either autochthonous MMTV-PyMT breast carcinoma cells or orthotopically implanted POSTN-deficient breast carcinoma cells, was also attenuated with the POSTN^−/−^ background as compared to the control POSTN^+/+^ background [30]. Moreover, the attenuated metastatic potential was associated with decreases in both CD24^+^CD90^+^ CSC-enriched cancer cell proportions and mammosphere formation, and the addition of POSTN reversed the decreased mammosphere formation in MMTV-PyMT POSTN^−/−^ tumour cells. Furthermore, stromal POSTN was shown to interact with Wnt ligands to augment the presumed signalling activity. Taken together, these findings demonstrated that CAF-produced POSTN in the lung drives metastatic colonisation of disseminated breast carcinoma cells via induction of Wnt signalling and thus the CSC trait.

TN-C, as a crucial component of the metastatic environment, is also an appealing target for new therapeutic strategies. FSP-1^+^ stromal fibroblasts that contribute to producing TN-C were eradicated in transgenic mice upon expression of viral thymidine kinase under the control of the FSP-1-promoter and with ganciclovir treatment [77]. As a result, pulmonary metastases of orthotopically and intravenously implanted 4T1 breast carcinoma cells were significantly decreased [77]. Similar results were observed in TN-C-null mice which had received these cancer cells intravenously. Collectively, these findings raise the possibility that CAFs not only provide carcinoma cells in tumour with CSC and invasive properties but also serve as a niche that can support their metastatic colonisation at distant sites.

## 5. Therapeutic Targeting of the Key Signalling Pathways Associated with CAFs

**Figure 1:**
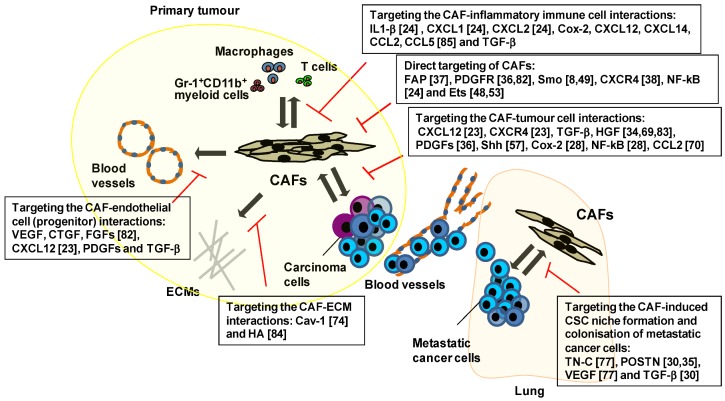
Schematic representation of approaches targeting interactions of CAFs with other tumour-constituting cells during tumour progression. Targeting CAFs themselves and disturbing their interaction with other cell types and/or ECM within tumours have shown promising anti-cancer effects in different experimental mouse tumour models. Inhibiting the expression and/or activity of FAP [37,80,81], PDGFR [36,82], Smo [8,49], CXCR4 [38], NF-κB [24] or Ets [48,53] in CAFs attenuated tumour growth, decreased angiogenesis, improved intratumoural drug delivery and/or restored anti-tumour immune responses. Blocking interaction between CAFs and carcinoma cells through CXCL12 [23], CXCR4 [23], TGF-β, HGF [11,34,69,83], PDGFs [36], Shh [57], Cox-2 [28], NF-κB [28] or CCL2 [70] also attenuated tumour growth, progression, intratumoural drug delivery and/or innate drug resistance. Moreover, targeting VEGF, CTGF, FGFs [82], CXCL12 [23], PDGFs orTGF-β, which have been implicated in CAF-endothelial cell (progenitor) interactions, showed inhibition of neoangiogenesis and vessel stabilty. Inhibitingcaveolin-1(Cav-1) [74] and HA [84] mainly produced by CAFs, also suppressed tumour invasion and metastasis by affecting their interaction with ECM. Targeting CAF-induced inflammation and immunosuppression via inhibition of IL-1β [24], CXCL1 [24], CXCL2 [24], Cox-2, CXCL12, CXCL14, CCL2, CCL5 [85] or TGF-β was also effective for preventing neoangiogenesis, as well as tumour growth and progression. Furthermore, targeting TN-C [77], POSTN [30,35], VEGF [77] or TGF-β [30] mediating the CAF-induced CSC niche formation, attenuated metastatic colonisation in the lung.

Targeting the tumour-associated stroma is believed to be essential for the development of new and effective cancer therapies [4,6,12,13,79]. Genes and signalling pathways mediating interactions of CAFs with other tumour-constituting cells, are also considered to hold promise as a therapeutic target (Figure 1, Table 2).

**Table 2:** Inhibitors targeting genes and the signalling pathways relevant to the CAF-based cancer therapy. FAP-based CAF-targeting therapeutic approaches using their neutralising antibodies [86,87,88,89], inhibitors [90,91,92], prodrugs [93,94] and DNA vaccine [81,95] showed attenuation in tumour growth via improving tumoural immune response in different experimental mouse tumour models. SDF-1-CXCR4, HGF-Met, Shh-Smo, PDGF-PDGFR and TGFβ-TGFβR signalling pathways or HA ECM protein have been reported to mediate the CAF-tumour(stroma)cellinteractions. Inhibition of these genes and signalling pathways using small molecule inhibitors [8,49,55,82,83,84,96,97,98,99] and neutralising antibodies [10,23,49,54,96,98,100] has shown attenuation in tumour growth, tumour progression and/or neoangiogenesis, as well as improvement in innate drug resistance, interstitial fluid pressure (IFP) and/or intratumoural drug delivery.

Genes and the signal pathways	Inhibitors	Effects
FAP	Sibrotuzumab^+ ^(an anti-FAP inhibiting antibody) [87,88]; FTPD * and FAP5-DM1 * [86 ] (anti-FAP antibodies conjugated to anti-cancer drugs); Val-boro Pro (Talabostat) ^+^ [90 , 92 ] and PT630 * [91 ] (FAP chemical inhibitors); Prodrugs ^+^ [93 , 94 ] DNA vaccine * [81 , 95 ]	Increased immune response
SDF-1-CXCR4 signalling	An anti-SDF-1 neutralising antibody * [23]	Decreased neoangiogenesis and tumour growth
HGF-Met signalling	GDC-0712 * (a MET small molecular inhibitor) [83]; NK4 * (an HGF antagonist) [96 ]; An anti-HGF neutralising antibody* [96 ]	Decreased innate drug resistance
Shh-Smo signalling	IPI-926 *, HhAntag * and MS-0022 * (Smo small molecular inhibitors) [8,49,55]; 5E1 * (a Shh neutralising antibody) [49 , 54 ]	Increased neoangiogenesis and improved intratumoural drug delivery
PDGF-PDGFR signalling	Imatinib * (a tyrosine kinase inhibitor) [82]; PDGF-C-neutralising antibodies * [10 ]	Decreased IFP and improved intratumoural drug delivery
TGFβ -TGFβ R signalling	An anti-TGFb neutralising antibody (1D11) *^,+^ [98,99,100]; A TGFbRI antagonist *^,+^ [97 , 98 , 99 ] PEGPH20 * [84]	Increased vascular permeability and improved intratumoural drug delivery
HA		Decreased IFP and improved intratumoural drug delivery

+ Clinical trial study, * Preclinical study.

It has been reported that the ability of these fibroblasts to remodel the ECM and generate tracks for carcinoma cell migration is associated with their collagen gel contractile ability [101]. A recent chemical screening study identified 3-hydroxy-3-methylglutaryl-coenzyme A reductase inhibitors, such as simvastatin and lovastatin, which can suppress the collagen gel contraction induced by CAFs. These inhibitors were also able to substantially attenuate SCC migration by disrupting the function of the Rab small G proteins [101]. Collectively, these observations indicate the potential therapeutic feasibility of targeting the ECM remodelling and SCC invasion driven by CAFs.

The activation of PDGFR signalling is known to be crucial for CAFs to conduct their tumour-promoting function [50,102]. PDGFs, which are mainly produced by carcinoma cells, stimulate proliferation and activation of CAFs by acting through PDGFRs [50,82]. PDGFR signalling promotes CAF functions that are relevant to tissue contraction leading to high interstitial fluid pressure (IFP). The resulting vascular collapse also creates a substantial barrier against drug delivery [36,103,104]. A recent drug screening study of 160 kinase inhibitors which used cultured primary human lung CAFs, identified several PDGFR kinase inhibitors that significantly suppressed the proliferation of these cells [105]. Among these PDGFR kinase inhibitors, dasatinib blocked CAF proliferation most efficiently, without inducing cell death at concentrations below 100 nM. Notably, other *in vivo* studies using tumour xenografts and transgenic tumour mouse models also showed that inhibition of PDGFR signalling in CAFs facilitates intratumoural drug delivery [106,107]. The inhibition of PDGFR signalling using the selective PDGF receptor kinase inhibitor STI571 and an antagonistic PDGF-B oligonucleotide aptamer thus decreased IFP and facilitated drug delivery, thereby increasing the efficacy of chemotherapy and attenuating tumour growth [106,107].

In addition to PDGF-PDGFR signalling, Hh-Smo signalling also plays important roles in modulating intratumoural drug delivery. Hh ligands, which are released from carcinoma epithelial cells, induce Smo activity via binding to the PTCH1 receptor expressed on the surrounding stroma, as mentioned earlier [49]. Effects of chemical inhibitors suppressing Smo signalling activity in stromal fibroblasts on drug delivery were also investigated in tumour xenografts and transgenic tumour mouse models [8,49,55]. Notably, inhibition of Hh-Smo signalling mediating the tumour-stromal interaction was shown to facilitate efficient intratumoural delivery of cytotoxic drugs [8,49,55].

An ECM component known as hyaluronan (also called hyaluronic acid) abundant in tumour stroma is a simple polysaccharide molecule composed of repeating disaccharide units of alternating *N*-acetylglucosamine and glucuronic acid [108]. The dynamic turnover of hyaluronan is tightly regulated by its synthesis and degradation, and excessive hyaluronan production is associated with poor outcomes of human carcinomas. The high molecular-weight hyaluronan and its degradation fragments enriched in the tumour microenvironment play important roles in promoting innate immune responses through interacting with their receptors, such as toll-like receptors, CD44 and versican [108]. A recent study demonstrated a new role of hyaluronan in forming a physical barrier against chemotherapeutic agents [84]. It has thus been shown that enzymatic depletion of this ECM protein prior to gemcitabine treatment inordinately increased cytotoxic efficacy in an autochthonous murine pancreatic ductal adenocarcinoma model. Reduced IFP and increased microvasculature were also responsible for an increase in cytotoxic efficacy, thereby resulting in the suppression of tumour growth and metastasis. These findings suggest that ablating specific stromal proteins and their related signalling pathways can render tumours profoundly vulnerable to conventional chemotherapy in experimental animal models.

## 6. Therapies Targeting CAF-Mediated Anti-Immune Responses and Anti-Drug Resistance

Immune suppressive roles of the tumour microenvironment that prevent suppression of tumour growth have widely been accepted [109,110]. The tumour-associated stroma includes various cell types relevant to immune suppression, such as Gr-1^+^myeloid-derived suppressor cells, CD4^+^Foxp3^+^ T regulatory cells and MSCs. Several recent studies have also indicated that CAFs contribute to tumoural immune suppression [80,81,95].

FAP is a type II transmembrane protein that functions as a post-prolyl protease. The expression of this protein is also detected on the cell surfaces of activated fibroblast populations of CAFs. FAP-null mice in fact showed a decrease in the tumourigenicity of endogenous K-rasG12D-driven lung carcinoma cells and syngeneic colon tumour cells [91]. Targeting CAFs on the basis of their FAP expression has also been achieved by employing oral immunisation with an attenuated Salmonella strain expressing FAP in immunocompetent mouse tumour xenograft models [81]. The orally administered DNA vaccine notably suppressed neoangiogenesis, tumour growth and metastasis of orthotopically injected breast carcinoma cells [81]. This decreased tumour progression was attributed to a deceased amount of type 1 collagen production and increased recruitment of CD8^+^ T cells into the tumour. Moreover, treatment with the DNA vaccine together with doxorubicin substantially increased intratumoural uptake of the cytotoxic drug and prolonged lifespans of the vaccinated mice [95]. Collectively, these findings demonstrate the important roles of CAFs in modulating tumoural immune suppression and the aforementioned intratumoural drug delivery.

The biological roles of FAP-positive (FAP^+^) CAFs were further studied using a transgenic mouse expressing the diphtheria toxin receptor gene under the control of the 217 kb FAP gene promoter region cloned into a bacterial artificial chromosome [37]. FAP^+^ CAFs were selectively eliminated by administering diphtheria toxin to transgenic mice bearing Lewis lung carcinoma cells expressing ovalbumin that rendered the cells immunogenic. As a result, the subcutaneous immunogenic tumour showed substantially attenuated growth. The decreased tumour growth was also mediated by induction of rapid hypoxic necrosis of both cancer and stromal cells without altering the T cell proportions present in the tumour. Moreover, neutralising antibodies against tumour necrosis factor-α or interferon-*r* restored tumour growth inhibition by ablating FAP^+^ CAFs, indicating essential roles of these fibroblasts in modulating carcinoma cell responses to the cytotoxic effects of both cytokines. Taken together, these data support the notion that FAP^+^ CAFs play immunosuppressive roles in promoting the tumourigenesis of immunogenic tumour cells.

To extend the FAP expression-based CAF targeting approach sufficiently to allow a preclinical study, FAP-blocking antibodies were employed in different tumour xenograft mouse models [80,86,89]. A monoclonal anti-FAP antibody was covalently linked with DM1, a tubulin-binding maytansinoid with antimitotic activity, known as FAP5-DM1 [86]. This antibody substantially attenuated the growth of tumour xenografts obtained from various cancer cell lines implanted into recipient mice with no evidence of toxicity. However, a clinical trial using an anti-FAP antibody (m-Ab F19; sibrotuzumab) showed no significant efficacy for the treatment of metastatic colorectal cancer [87,88].

FAP expression is detected in a major CAF population, and its unique enzymatic activity allows for the development of FAP-activated pro-drugs designed as potent cytotoxic agents [80,93,94]. These pro-drugs, which are composed of cytotoxic agents coupled with a peptide carrier containing a FAP cleavage site, remain inactive when systemically delivered. They are then proteolytically activated by FAP expressed on CAFs within the tumour. Once activated, it is anticipated that the resulting cytotoxins will specifically target any nearby cells that are present within regions of the drug activation, including tumour cells and any stromal cells. Notably, doxorubicin and protoxin conjugated with a FAP-specific dipeptide exhibited increased anticancer efficacy without serious adverse effects [93,94]. These CAF-based tumour-targeting/anti-cancer therapeutic approaches will be further validated in different xenograft and autochthonous tumour mouse models.

A number of human carcinomas initially sensitive to specific therapeutic approaches, are known to often become refractory to these drugs during treatment [111]. This is a very challenging problem to overcome in the clinical setting. The ability of the tumour-associated stroma to elicit innate drug resistance in tumours has also recently been demonstrated [7,11,83,96,112]. HGF, which is released by CAFs, promotes tumour cell migration and invasion [34]. This stromal growth factor was also shown to hinder the growth suppression of carcinoma cells by selective inhibitors. Thus, HGF treatment restored growth inhibition of epidermal growth factor receptor (EGFR)-mutant lung carcinoma cells by the EGFR tyrosine kinase inhibitor gefitinib [96] and that of BRAF-mutant melanoma cells by the BRAF inhibitors PLX4720 and PLX4032 [11,83]. This observed innate drug resistance was in fact mediated by Met receptor activation and downstream effectors, notably including MAPK and phosphatidylinositol-3-OH kinase (PI(3)K)-AKT signalling pathways [11,83,96]. Moreover, inhibition of either HGF or Met function resulted in reversal of stromal HGF-induced drug resistance. These findings, therefore, indicate the importance of stromal HGF-activated Met signalling in carcinoma cells that underlies innate drug resistance via MAPK and PI(3)K-AKT signalling. It would also be interesting to determine whether the tumour-associated stroma is involved in conferring stable, adaptive drug resistance in carcinoma cells, possibly via the acquisition of epi/genetic alterations during treatment.

## 7. Conclusions and Perspectives

It has become apparent that CAFs serve as a niche promoting tumour growth and progression by conferring invasive, metastatic and CSC phenotypes upon neighbouring carcinoma cells. There have been reports on a number of tumour-promoting signalling pathways that mediate the interactions between CAFs and different cell types existing within tumours. Targeting the CAF-orchestrated tumour-prone microenvironment should therefore be applicable to developing novel cancer treatments. This, however, remains a challenging task due to the presence of distinct CAF populations expressing α-SMA, TN-C, FAP, FSP-1 (also called S100A4) and/or PDGFR-α/β. Targeting a particular CAF population in genetically engineered mouse tumour models may not fully reveal the biological roles of all CAF populations during tumourigenesis. Moreover, these molecular markers can’t distinguish activated fibroblasts within tumour from these cells also present in non-cancerous tissues. This would thus make the specific targeting of the tumour-associated fibroblasts *in vivo* difficult without affecting activated fibroblasts in non-cancerous tissues. Tumour xenograft models using the co-implantation with carcinoma cell lines are useful for evaluating the tumour-promoting functions of entire CAF populations; however, such assays rely to a certain extent on non-physiological characteristics ostensibly acquired by the carcinoma cells during their long-term propagation in culture. Recently, patient-derived tumour xenografts (PDTX) engrafted into immunocompromised mice have been documented to reflect tumour histopathology, growth and disease outcomes [113,114]. It is thus plausible that the preclinical xenograft mouse model can be employed by the PDTX-derived primary human carcinoma cells and human CAFs, each of which has been marked by different reporter plasmids. During therapeutic trials, real-time monitoring of these cells by *in vivo* imaging technology and extracting them for subsequent *in vitro* analyses will allow us to evaluate the efficacy and specificity of CAF-targeting anti-tumour therapies. Further implantation of human immune cells may be also considered in efforts to render the PDTX mouse partially immunocompetent [115].

Several multi-targeted anti-angiogenic tyrosine kinase inhibitors (e.g., sorafenib) against vascular endothelial growth factor receptors (VEGFRs), PDGFRs, fibroblast growth factor receptors (FGFRs) and/or epidermal growth factor receptors (EGFRs), showed a greater anti-tumour efficacy than single-target drugs in patients, likely reflecting the facilitated acquisition of drug resistance [116]. Recent network analyses that monitored gene signalling pathways, gene expression profiles and cell phenotypic responses, also revealed that pre-treatment with an EGFR inhibitor, such as erlotinib, substantially sensitised breast carcinoma cells to apoptotic signalling induced by genotoxic drugs [117]. This greater efficacy was attributed to an increase in the DNA damage response mediated by caspase-8 cleavage that was initially inhibited by EGFR-dependent oncogenic signalling. Similarly, the CAF-induced oncogenic signalling pathways in carcinoma cells believed to influence tumour progression and the efficacy of genotoxic drugs, would also be potential therapeutic targets. The above-described studies demonstrated that targeting molecules and signalling pathways active in tumour-associated stroma successfully inhibited and even reversed tumour progression and improved the efficacy of chemotherapy in experimental tumour mouse models [8,36,49,84]. Further development of preclinical CAF-targeting approaches will hopefully help to improve the current conventional cytotoxic agents used for cancer chemotherapy.
